# Supplementary material for: Learning to predict future locations with internally generated theta sequences
Source: PLoS Comput Biol. 2023 May 12;19(5):e1011101. doi: 10.1371/journal.pcbi.1011101 (PMC10208522; doi:10.1371/journal.pcbi.1011101)
Supplement: S1 Text — This supplementary text contains a mathematical derivation showing how place field sizes and phase precession slopes can remain constant despite changes in instantaneous running speed if the speed of propagation of the activity bump within theta cycles is made dependent on instantaneous running speed. (PDF) [file pcbi.1011101.s001.pdf]

S1 Text

Making place field sizes invariant of instantaneous speed

In behavior-dependent sweeps, the position represented by the place cell population at a certain time point, $r(t)$, should be equal to the real position of the animal at that time step, $x(t)$, plus the distance the animal would cover if it ran forward at its typical speed through each location for a certain amount of time that increases across the theta cycle. In particular, assuming that the theta sweep spans the whole theta cycle of period T, the extent of the temporal look-ahead would be $\frac{t-t_n}{T}\tau_\theta$, where t_n is the beginning of the current theta cycle, and τ_θ is the maximum extent of the temporal look-ahead. If we assume for simplicity that the typical speed is constant across the relevant portion of the environment, we can express $r(t)$ as:

$$r(t) = x(t) + \frac{(t-t_n)}{T}\tau_\theta \bar{v} \tag{1}$$

Note that this would keep place field sizes constant despite changes in instantaneous running speed. That is because place field sizes, s, are proportional to the maximum look-ahead distance, which does not depend on the current running speed:

$$s \propto r(t_n+T) - x(t_n+T) = \tau_\theta \bar{v} \tag{2}$$

Taking the derivative of Eq. 1 with respect to time, we obtain the speed of the theta sweep:

$$v_\theta(t) = v(t) + \frac{\tau_\theta}{T}\bar{v} \tag{3}$$

To translate this speed in physical space to speed in the network, we need a measure of the density, d, which indicates the number of units in the network tiling each unit of space. Assuming an ideal mapping between physical and neural space, the density could be obtained by dividing the number of units eligible for plasticity traversed per unit of time, u_0, which is some constant defined by the internal network dynamics, by the amount of space traversed per unit of time at the average running speed:

$$d = \frac{[units]}{[space]} = \frac{\frac{[units]}{[time]}}{\frac{[space]}{[time]}} = \frac{u_0}{\bar{v}} \tag{4}$$

Multiplying $v_\theta(t)$ with this density, we obtain $s_\theta(t)$, the speed of propagation of theta sweeps in neural space:

$$u_\theta(t) = u_0\left(\frac{\tau_\theta}{T} + \frac{v(t)}{\bar{v}}\right) \tag{5}$$

Thus, the speed of theta sweeps in the network is proportional to a constant plus the ratio between instantaneous and average running speed at those locations. In realistic conditions, the first term is significantly larger than 1 whereas the second term fluctuates around 1. The first term corresponds to activity propagation in our model which is driven by the intrinsic network dynamics. The second term with its dependency on instantaneous running is not accounted for in the current implementation of our model and would have to be added to approximate behavior-dependent sweeps more closely and keep place field sizes constant. Moving bump attractors with velocity-dependent inputs has been implemented previously in models of path integration [1, 2].

References

[1] B. L. McNaughton, F. P. Battaglia, O. Jensen, E. I. Moser, and M.-B. Moser, "Path integration and the neural basis of the 'cognitive map'," *Nature Reviews Neuroscience*, vol. 7, pp. 663–678, Aug. 2006.

[2] Y. Burak and I. R. Fiete, "Accurate Path Integration in Continuous Attractor Network Models of Grid Cells," *PLOS Computational Biology*, vol. 5, p. e1000291, Feb. 2009.
