# Supplementary material for: Learning to predict future locations with internally generated theta sequences
Source: PLoS Comput Biol. 2023 May 12;19(5):e1011101. doi: 10.1371/journal.pcbi.1011101 (PMC10208522; doi:10.1371/journal.pcbi.1011101)
Supplement: Attachment — Submitted filename: PCB review.docx [file pcbi.1011101.s002.docx]

The authors proposed a computational model for explaining their previous findings published in eLife, where they showed that as the animal runs faster, place fields are wider and phase precession is shallower in those areas. Specifically, the model consists of a continuous attractor network with short-term synaptic facilitation and depression that internally generates theta sequences that advance at a fixed pace. Spatial locations are then mapped onto the active units via modified Hebbian plasticity, so that neighboring units end up representing spatial locations further apart where animals run faster, reproducing their earlier experimental results. The model also explains over-representation of locations when animals slow down in those areas. The novel contribution of this paper is a computational model of position mapping through Hebbian learning, which provides a way of hippocampal information coding that arises from the interplay between sensory input and predefined network dynamics.

Below are the major/minor points I would love the authors to answer.

**Major points:**

- The function of different parts of the model is not well introduced. In the previous Tsodyks’ model, the activity bump is driven by the external moving input, which travels faster in individual theta cycles either due to the asymmetric connection (Tsodyks et al, 1996) or STD (Romani and Tsodyks, 2015). However, the authors pointed out that the activity bump reappeared slightly ahead of where the last one formed. Can the authors explain the role of the external input in this model and if the bump's location is driven by the external input or by internal mechanisms such as STD/STF? Additionally, can the authors provide information on how the model's hyper-parameters were tuned to match the intrinsic speed of the bump with the external speed of the artificial animal (if it is the case)?
- The authors mention that Hebbian learning shapes the density of spatial coding of individual neurons, but do not specify how this affects the speed of the activity bump in the neuronal space. Can the authors explain if the activity bump always travels with a constant speed in neuronal space regardless of the animal's running speed?
- Theta frequency is known to vary with running speed. However, in the continuous attractor, the rhythmic input is set as a fixed frequency. Can the authors explain how this may affect the phase precession slope and if the model's conclusions still hold in cases where theta frequency is varied with running speed.
- I am confused by the instantaneous running speed in their analysis (specifically in Fig. 4E&F). Can the authors explain what is meant by instantaneous speed and how it differs from the non-instantaneous speed (or the characteristic speed mentioned in the authors' previous eLife paper)? Additionally, can the authors provide insight on how their conclusions would change if the analysis was done using for example, the top and bottom 20% of running speeds instead of the top and bottom half of instantaneous speeds (in the plot of Maurer et al. (2012) (Fig. S1), they used low (20-40cm/s) and high (80-100cm/s) velocity bins to show place field size is invariant against running speed)?
- Is there any experimental evidence supporting the internal structure of place cells in CA3 as a continuous attractor network (CAN)? The preplay papers only indicate internally generated sequences in the HPC, not a CAN.

**Minor points:**

- The title is confusing. The network model with STD/asymmetric connections (Tsodyks’ model) can internally generate bump sweeps, which does not require any learning. Moreover, there seems no prediction of future locations, since it is a 1D linear track environment (see Johnson and Redish, 2007, JNS). Can the authors provide a clearer title that reflects the novel contribution of the paper?
- Fig. 2B is not clear. Better to split into two plots with one showing the internally driven sequence and the other showing the externally driven sequence.
- Fig. 2B is not clear. It would be beneficial to split the figure into two plots, one showing the internally driven sequence and the other showing the externally driven sequence, to improve clarity.
- Why is the spatial input also rhythmic? What if it is a constant input with changing location?
- Could the change of the place field size with running speed be an analysis artifact? The usual way of calculating the place field size is to calculate the firing rate at each spatial bin and then cut a threshold to determine the firing field. When animals run slower, the firing rate is lower, then cutting the same threshold as when the animals run faster will lead to a smaller place field.

Overall, the computational model is promising. However, the results appear to be a direct replication of previous work and may require further verification. My recommendation is for a major revision.

I hope that the authors will find my feedback helpful in improving their work.
