# Supplementary material for: Learning to predict future locations with internally generated theta sequences
Source: PLoS Comput Biol. 2023 May 12;19(5):e1011101. doi: 10.1371/journal.pcbi.1011101 (PMC10208522; doi:10.1371/journal.pcbi.1011101)
Supplement: Attachment — Submitted filename: response to reviewers.odt [file pcbi.1011101.s003.odt]

Dear reviewers, Dr. Bush and Dr. Graham, 

We would like to express our gratitude for your thoughtful, extensive and constructive feedback on our manuscript. In the following, we detail point-by-point how we have improved our manuscript based on your suggestions and concerns.
________________________________________________________________________
Reviewer #1
Major points:
1, The function of different parts of the model is not well introduced. In the previous Tsodyks’ model, the activity bump is driven by the external moving input, which travels faster in individual theta cycles either due to the asymmetric connection (Tsodyks et al, 1996) or STD (Romani and Tsodyks, 2015). However, the authors pointed out that the activity bump reappeared slightly ahead of where the last one formed. Can the authors explain the role of the external input in this model and if the bump's location is driven by the external input or by internal mechanisms such as STD/STF? Additionally, can the authors provide information on how the model's hyper-parameters were tuned to match the intrinsic speed of the bump with the external speed of the artificial animal (if it is the case)?
This point might have been confusing because, in our model, the activity bump is driven by different factors at different stages of learning. Initially, the activity bump is entirely driven by preconfigured internal mechanisms, since the connections from the weakly spatially tuned inputs to the to-be-place-cells are not yet learned. The network is preconfigured to generate sequential activity on two different time scales via different mechanisms. Within each theta cycle, the activity bump moves forward due to slightly asymmetric recurrent connections and short-term depression. From one theta cycle to the next, an interplay between short-term depression and short-term facilitation advances the initial position of the bump. At this stage, the model operates similarly to the one described by (Wang et al., 2015) (their Fig. 3c), which was actually proposed as a model of time cells. 
The network dynamics changes after learning (i.e., after about 60 seconds in our simulations), due to the increased weights between inputs and place cells. Most importantly, the initial position of the bump at the beginning of each theta cycle becomes sensitive to the spatial inputs. This influence can be seen as pulling the activity bump from the units in the network in which it would have formed based only on its internal mechanisms, to the units in the network that have become associated with the current position in space. In particular, if the animal runs faster than average, the units in the network associated with the current position of the animal will be further ahead than those that would have become active based only on the network’s internal mechanisms, and so the spatial inputs will pull the activity bump forward. Conversely, if the animal runs slower than average, the spatial inputs will pull the activity bump backwards. On average, though, the units in the network associated with the current position will coincide with the units that would have become active based on the network’s internal mechanisms. This rough match is the direct result of the Hebbian learning process, rather than the tuning of network hyper-parameters. 
We modified the text to clarify these points. 

2, The authors mention that Hebbian learning shapes the density of spatial coding of individual neurons, but do not specify how this affects the speed of the activity bump in the neuronal space. Can the authors explain if the activity bump always travels with a constant speed in neuronal space regardless of the animal's running speed?
As described in our reply to the first question, it is important to distinguish between two different time scales. Within theta cycles, the speed of the bump is constant because it only depends on the intrinsic dynamics, which is driven by the asymmetry of recurrent weights and short-term depression. However, after learning the jump in the initial position of the bump from one theta cycle to the next depends on both intrinsic dynamics and external spatial inputs. This introduces the speed-dependence of spatial coding, which we described above, and have now explained in the manuscript.

3, Theta frequency is known to vary with running speed. However, in the continuous attractor, the rhythmic input is set as a fixed frequency. Can the authors explain how this may affect the phase precession slope and if the model's conclusions still hold in cases where theta frequency is varied with running speed.
If theta frequency increases while all other parameters of our model are left unchanged, then theta sweeps would be shorter, resulting in steeper theta phase precession and smaller place fields. However, the increase in theta frequency with speed appears to be relatively modest (1-2 Hz within the range of speeds assessed in experiments (Shen et al., 1997; Sławińska & Kasicki, 1998; Kennedy et al., 2022; Dunn et al., 2022)). Most importantly, there appear to be compensatory mechanisms in place such that the change in theta frequency with speed does not affect the phase vs. position relationship of cell’s spikes (Huxter et al., 2003; Schmidt et al., 2009; Parra-Barrero et al., 2021). One could easily imagine what those compensatory mechanisms would look like in our model. Essentially, the speed of propagation of the activity bump would also need to be made proportional to running speed. This could be mediated by the increase in firing rate that is also known to occur with speed. However, taking these effects into account would increase the complexity of the model, presumably without changing the overall principles by which it operates and which are the focus of this study, and therefore we chose not to include them at this stage. 
We added this explanation to the Discussion section where we discuss the effect of speed on hippocampal activity.

4, I am confused by the instantaneous running speed in their analysis (specifically in Fig. 4E&F). Can the authors explain what is meant by instantaneous speed and how it differs from the non-instantaneous speed (or the characteristic speed mentioned in the authors' previous eLife paper)? Additionally, can the authors provide insight on how their conclusions would change if the analysis was done using for example, the top and bottom 20% of running speeds instead of the top and bottom half of instantaneous speeds (in the plot of Maurer et al. (2012) (Fig. S1), they used low (20-40cm/s) and high (80-100cm/s) velocity bins to show place field size is invariant against running speed)?
By instantaneous speed we mean the speed at which the simulated animal / agent is running in any given moment, as opposed to the mean (or average or ‘characteristic’ in the eLife paper) speed of the agent in each portion of the environment. In particular, in the analyses in Fig. 4E&F, we calculated place fields using only time steps with instantaneous running speed above or below the median speed at each spatial bin. We have now rerun the analyses using the top and bottom 20% of speeds at each spatial bin, obtaining very similar results. The figure now shows the new results. 

Is there any experimental evidence supporting the internal structure of place cells in CA3 as a continuous attractor network (CAN)? The preplay papers only indicate internally generated sequences in the HPC, not a CAN.
There is a long tradition of characterizing CA3 place cells as continuous attractor networks (e.g., Samsonovich & McNaughton, 1997; Azizi et al., 2013; Romani & Tsodyks, 2015; Corneil & Gerstner, 2015; Knierim & Neunuebel, 2016; Rolls, 2017; Laptev & Burgess, 2019). To us, some of the most convincing evidence in favor of attractor dynamics in CA3 involves the presence of abrupt transitions between representations of different learned environments (Colgin et al., 2010; Jezek et al., 2011). 

Minor points:
1, The title is confusing. The network model with STD/asymmetric connections (Tsodyks’ model) can internally generate bump sweeps, which does not require any learning. Moreover, there seems no prediction of future locations, since it is a 1D linear track environment (see Johnson and Redish, 2007, JNS). Can the authors provide a clearer title that reflects the novel contribution of the paper?
We believe the title is fitting. We model behavior-dependent sweeps, which extend to the location that animals would reach a fixed time interval in the future assuming they ran at the speed that is characteristic of each part of the environment. In other words, during the theta cycle, the place cell population represents locations which are forward of the agent’s/animal’s current location. We think that it is reasonable to call this a prediction of future locations, regardless of whether it is in 1D or not. Furthermore, it is only through learning that our model develops these behavior-dependent sweeps; without learning there would not be a consistent relationship between network activity during a theta cycle and the spatial positions of the agent at different time points. Therefore, the model is learning to represent, for any given location, the other locations that will be reached on average a certain time interval in the future, and then generating theta sequences that go from one to the other. 

2, Fig. 2B is not clear. Better to split into two plots with one showing the internally driven sequence and the other showing the externally driven sequence.
3, Fig. 2B is not clear. It would be beneficial to split the figure into two plots, one showing the internally driven sequence and the other showing the externally driven sequence, to improve clarity.
We have implemented this suggestion and agree that it makes the figure clearer. 

4, Why is the spatial input also rhythmic? What if it is a constant input with changing location?
We limited the influence of the input on the network to the beginning of each theta cycle so that we could have clearly differentiated phases for (1) encoding the current location and (2) predicting upcoming locations, in line with the proposal that there exist different phases for encoding and retrieval (Hasselmo et al., 2002). Not doing so tends to make the learning unstable. To intuit why this happens, consider the following example. Let us say the first cell in the sequence becomes associated with location x = 0. Because of the theta sequence, cells 2, 3 and 4 will activate in rapid succession before the location of the animal has changed significantly. Therefore, if learning is allowed to happen throughout the whole theta cycle, those cells would also become associated with location x ≈ 0. Then, next time the animal is at x = 0, the spatial inputs would initiate an activity bump not at the first cell, but perhaps at cell number 2 or 3, which is in the middle of all of the cells associated with that location. Then again, cells 4, 5 and 6 will activate and become associated with roughly the same location, shifting the position of the bump associated with that location even further, and so on. 

5, Could the change of the place field size with running speed be an analysis artifact? The usual way of calculating the place field size is to calculate the firing rate at each spatial bin and then cut a threshold to determine the firing field. When animals run slower, the firing rate is lower, then cutting the same threshold as when the animals run faster will lead to a smaller place field.
As mentioned in our response to Q3, in our model the activation level (equivalent to the firing rate) is not dependent on running speed, so the effect that the reviewer hypothesizes could not have occurred in our model results. Furthermore, we calculated place field sizes using a threshold that is proportional to the peak firing rate of each place field. Therefore, even if there were variations in peak firing rates, they would not affect place field sizes. This is also what we did in our eLife paper when analyzing the experimental data.
Reviewer #2
1. The authors make much of the argument that prior research has suggested that sweeps start from behind the animal and proceed to locations ahead of them. If one were to ask a bipedal animal where they are in Cartesian coordinates with respect to the floor, they would most-likely point to their feet. In a quadrupedal animal, it is inherently more complicated to ask the animal “where exactly are you?” Do they consider their position to be their head (where that investigator always decodes the position – resulting in sweeps initiating from behind) or the other extreme, their tail (where all sweeps would be prospective)? Perhaps something in between? Without explicitly knowing where the animal considers to be their “absolute location,” the emphasis on where the sweep initiates seem forced and artificial.
We agree that there is some ambiguity as to which part of their body animals like rats use as their reference point when determining their location. If the animal used a different reference point than the LED placed on its head, one should apply a correction to the tracking data before calculating firing rate maps and decoding positions from population activity. For instance, if it was determined that rats use their nose as their reference point, then the positions measured in experiments should be shifted forward; and if they use their center of mass as their reference point, then positions should be shifted backwards. Since no such corrections are usually applied, the experimental position data might be wrong and shifted relative to the true reference point. However, that shift is problematic only if we tried to align data with physical locations. For instance, if we computed the firing maps when the animal was running in two opposing directions and then tried to compare them (e.g. whether they are aligned), then the shifts would have to be taken into account because they point in opposing directions (e.g., Muller & Kubie, 1989). Otherwise, we might conclude that there was a systematic misalignment of place fields in two opposing directions, even if in reality they are perfectly aligned if the correct reference point is used. However, since we analyzed the experimental data in our previous eLife paper for one running direction only, and other studies on theta phase precession and sequences do, too, any shift due to the reference point would not affect the results. Since the shift is the same for all positions, all the usual findings regarding theta sweeps would still hold. Theta sweeps would appear to start behind the rat’s reference point (wherever that is) and reach ahead of it. In the example mentioned by the reviewer where rats take their tails as reference points, tracking data would have been shifted to reflect this, and the decoded sweeps would start behind the tail and reach ahead of it.  
2. There is reason to believe that, when animals firsts enter an environment, all known speed modulations are absent (Environmental novelty is signaled by reduction of the hippocampal theta frequency; Jeewajee et al., 2007). This is potentially problematic as it would suggest that the physical space mapping in figure 1C could be uniformly distributed in a manner that matches the neuronal space (rather than clustered at the end of the tracks). Otherwise, the authors would need to account for why the uneven distribution occurs a priori to when the rat knows its going to run fast or slow at a certain location.
We are not entirely sure we understand the reviewer’s perspective here. To make sure there is not misunderstanding, our model makes no a priori assumptions about where cells in the network will develop place fields. The distribution of place fields emerges entirely through the interplay between inputs (driven by behavior), intrinsic dynamics and Hebbian plasticity. Fig. 2C shows the uneven distribution after learning with increasing and then decreasing speed along the track. If running speeds were constant across the environment, place fields would be distributed uniformly and their properties would be uniform as well. Perhaps the reviewer is suggesting that running speed profiles are very different when animals are introduced to a novel environment compared to when the environment has become familiar. However, since learning in our model takes a certain amount of time (and never stops), even if animals ran with a very uncharacteristic speed profile the first time they explore a novel environment, the learned mapping could still reflect the more stereotypical behavior animals manifest later. 
We would also like to address the slight reduction in theta frequency in novel environments reported by Jeewajee et al. We think there are reasons to believe that the theta phase code (the relationship between position and theta phase, which determines phase precession slope and theta sweep length) remains largely unperturbed by changes in theta frequency. The theta phase code remains constant despite changes in running speed that presumably change theta frequency (Huxter et al., 2003; Geisler et al., 2007; Schmidt et al., 2009; Parra-Barrero et al., 2021). Phase differences between pairs of cells also remain constant despite changes in theta frequency brought about by cooling the medial septum (Petersen & Buzsáki, 2020), and in bats, phase-position relationships are constant even in the absence of regular theta oscillations (Eliav et al., 2018). We added a discussion of the speed dependence of theta frequency in the revised manuscript.

3. In a recent paper, the author’s assumption that speed modulates theta sequences is directly challenged (Hippocampal place cell sequences differ during correct and error trials in a spatial memory task; Zheng et al., 2021). Zheng and colleagues state that as the running speed at the stopping point was the same in both successful and unsuccessful trials, differences in running speeds were not the cause of the differences in sequence slopes between successful and unsuccessful trials. How do the authors reconcile these results with their current manuscript? Certainly, addressing the Zheng results in the current manuscript seems pertinent.
This is a good point. There is also a study that shows longer theta sweeps when traveling to positions further away (Wikenheiser & Redish, 2015). Note, however, that the effects shown by both of these studies are relatively small compared to the effect of mean running speed we showed in our previous publication. Therefore, we believe that mean running speed is a major (or possibly, the major) determinant of theta sweep length, but certainly not the only one. We have added a paragraph to the Discussions to reflect this. 

4. While it is widely accepted that speed (with the manuscript described in point 3 above being among the outliers) is the factor responsible for setting the size of place fields/slope of precession/ theta sequences (effectively, all being different measures of the same phenomenon), placing objects has been found to result in a dramatic change in both place field size and phase precession slope (The Influence of Objects on Place Field Expression and Size in Distal Hippocampal CA1; Burke et al., 2011). These data also challenge the idea that speed is the only pertinent factor. Moreover, one could use these results to explain the results in figure 5. Leaving a goal location, the animal is quite confident about their location as they are departing a food reward. They begin to traverse a cue poor region of the track, resulting in higher uncertainty/larger sweeps. However, as they approach the goal, they can see the end of the track, and the reward and fields get small as the rat decreases the speed and approaches the next reward.
The results by Burke et al. are very interesting. However, note that in their experiments,  animals run slower when objects are present, especially at the beginning, presumably when place fields are being established (their Supporting Figure 2). Thus, it’s unclear whether the effects they report are independent of the differences in running speed or not. 
We would also like to note that in our previous publication, we considered the possibility outlined here that place cell activity depends on the distance to the nearest wall, rather than mean running speed. Mean running speed, however, was found to uniquely account for a larger portion of the variance. In any event, we acknowledged that further experimentation would be required to fully dissociate the effect of these variables, and we do not claim that speed is the only factor driving place field properties; it is possible that objects or boundaries contribute as well. We now mention this possibility in the Discussion. 

5. The above explanation seems to be as tenable as the discussion of acceleration. The discussion on acceleration seems almost like an afterthought, with no citations. While there is data from Kropff et al. that offers it is the driving factor of theta frequency, running counter to the mass of prior literature (Frequency of theta rhythm is controlled by acceleration, but not speed, in running rats; 2021), these data have been called into question by Kennedy et al. (A Direct Comparison of Theta Power and Frequency to Speed and Acceleration; 2022). The authors seem to have two pathways in addressing this – either explicating the issue, wading into the conflict, or not mentioning acceleration as it doesn’t seem directly relevant. I would suggest the latter.
In line with our reply to Reviewer 1’s 3rd question, and Reviewer 2’s 2nd question, we would like to point out again that there is no reason to suspect that changes in theta frequency become reflected in the phase vs. position relationship of cell’s spikes. Therefore, the controversy pointed out here regarding whether acceleration affects theta frequency or not is independent of the results of our model that we comment on. The model happens to produce slightly longer theta sequences when accelerating because theta sequences extend to the position the animal will reach on average a certain time interval in the future, and this will be further away when the animal is accelerating. We regard this as an interesting prediction that could be examined in experimental data. We believe the independence of these two effects is now more clearly reflected in the manuscript. 

6. Finally, I wish for the authors to consider a near-heretical idea. What If the hippocampal correlate is not space per se. And by extension, what If the velocity correlates are related to some other function that the brain is performing? For instance, Buzsaki and Llinas (2017) offer a unique perspective. That while it is easy to derive correlates between the brain's spatial-temporal activity and distance and duration measurements, these observations may be coincidental. They offer that it is not possible for the brain itself to directly sense space or time, concluding that, instead of searching for representations of space and time in the brain based on prior assumptions, it is important to investigate the brain mechanisms that lead to the formation of inferential, explanatory models. This perspective has been extended by Nadel and Maurer (2021), stepping away from space and time, to describe place cells as containers that serve to maintain stimulus equivalence or cognitive continuity from one moment to the next. From these perspectives, the authors observations could be reinterpreted under the assumption that the larger sweeps are related to regions without much change in space/time (velocity being the measure that relates these two things; e.g., cm/s) while smaller sweeps are instances where stimuli change relatively faster.
Thank you for bringing our attention to this. In fact, one of us has also proposed previously that the hippocampus is primarily about memory storage of any inputs it receives and not space per se (Cheng, 2013). There are some interesting differences, however, between the proposal by Maurer & Nadel and our model. In their proposal, the temporal rate of change of the population vector would depend on the temporal rate of change of the inputs (similarly, Tanni et al., (2022) propose that the spatial rate of change of the population vector depends on the spatial rate of change of the inputs). In many experiments, it is reasonable to assume that sensory stimuli are changing faster (with respect to space, and probably also with respect to time) near boundaries, where animals tend to run slower. 
In our simulations, however, the spatial rate of change of the inputs is approximately constant (because we generated weakly spatially tuned inputs homogeneously covering the whole track), and so the temporal rate of change is actually highest where the animals run faster. Therefore, in our model, the rate of change of the population does not track that of the sensory stimuli directly. Instead, the network learns to extract features that change at a rate defined by the network’s internal dynamics. We added a paragraph to discuss these issues. 

Reviewer #3
Major point 1: One claim is that reports of theta sequences starting from past positions are artifacts of a naive decoder and that the true sequences should start from the current animal position. This is a substantial statement. While this possibility has been identified, it would be helpful if a solution were proposed. In particular, in the authors' own simulated data, these sweeps also start from past positions. Could they create a decoding scheme such that these sequences would start from the current position? Ideally, this would not involve knowledge of spatial input, whose experimental measurement is often intractable. Would a decoder incorporating the theta phase of activity or the animal's speed be helpful?
This is an excellent point, and something we would like to explore further in future work. For the time being, we are happy to discuss some possibilities for how this might be done. Essentially, one would need to decode using firing rate maps corresponding to the place cells’ true place fields, where they receive spatial inputs. But like the reviewer points out, these are very challenging to measure directly. One possibility for estimating the locations where spatial inputs drive place cell activity could be based on the idea that place cells should fire most reliably (i.e., with the lowest variability) in these locations. That is because the external inputs at these locations act as anchor points for the activity of the cells, whereas firing outside of these locations is mediated by internal mechanisms that would presumably introduce some noise in the relationship between position and firing rate. Thus, one could try to find the portion of the measured place field (defined in terms of position and/or theta phases at which the spikes are emitted) that has the lowest trial-to-trial variability in firing rates. Decoding population activity using these portions of place fields should then result in the highest decoding precision (e.g., highest probability values in a Bayesian decoder). We added an explanation of this approach to the Discussions.

Major point 2: A worthwhile feature of this model is the implementation of learning over experience in a new environment (Fig. 3). Are there predictions concerning phase precession or theta sequences in this regard that have been or can be tested by experiments? Or is the learning process idiosyncratic to the model and is only meant to produce the appropriate learned network? The authors hinted at some features in their model, but a bit more thorough treatment would be beneficial. Their proposal that learning occurs in the associations with spatial inputs, and not the connections between place cells, is a major thesis of this work, and it seems natural that the learning mechanism in the model can be best tested through the analysis of learning in experiments.
The learning process is not only meant to produce the appropriate final network; we do regard it as approximating what could be happening in reality. At the same time,  not many studies have carefully examined the responses of place cells in a novel environment, and so we have not tuned the learning process to try to reproduce any particular finding. The main prediction of the model is that place cells would express place fields from the beginning, but their locations would shift back and forth or drift initially. Some studies have indeed shown that many place fields are present from the very first lap (Sheffield et al., 2017; Priestley et al., 2022), but that firing rate maps are initially unstable (Wilson & McNaughton, 1993; Frank et al., 2004). 
Our model also produces theta phase precession and theta sequences from the start. A study by Feng et al., 2015 has shown that cells indeed phase precess from the first lap, but only appear to start to participate in theta sequences from the second lap. However, animals run more slowly during the first lap (their Fig. 4a). According to our model, this would produce steeper phase precession, which they show (their Fig. 6c) and correspondingly, short theta sequences, which would be difficult to distinguish from no sequences. There is also evidence suggesting that the cells we have modeled, which belong to a predefined sequence, could constitute some sort of backbone onto which more cells are later recruited (Grosmark & Buzsaki, 2016), perhaps through behavioral timescale synaptic plasticity (Bittner et al., 2017).
We added these points to the Discussion.

Minor point 1: It is a bit difficult to see that activity bumps form at the location of highest (1-D)F in Fig. 3E. There is a band of high value that seems to lie right behind the formation location. Further clarification would be helpful.
The black outline in the figure corresponds to regions with an activation level above a certain threshold, therefore, the actual initiation of the bump occurs slightly before the black outline. We added this explanation to the Caption of Fig 3E.

Minor point 2: It would be helpful to include an explicit definition of "true place field" in Section 2.4. Although this term has been used by Sanders et al. (2015), it is not universal and can sound a bit overreaching.
We have defined the term more explicitly. 

Minor point 3: I could understand the Supplemental Material, but it would be helpful to motivate the calculation of theta sequence speed in network units earlier in the text. Also, it would be helpful to use different variable name in Eq. 11 or 14, since $s$ is used for different quantities (albeit with different subscripts).
We now introduce the calculation of speed in network units earlier in the main text. We also agree that the variable naming was misleading. We have changed the network propagation speed in Eqs. 13 and 14 to “u” to distinguish it from the place field size s.


References
Azizi, A. H., Wiskott, L., & Cheng, S. (2013). A computational model for preplay in the hippocampus. Frontiers in Computational Neuroscience, 7. https://doi.org/10.3389/fncom.2013.00161
Bittner, K. C., Milstein, A. D., Grienberger, C., Romani, S., & Magee, J. C. (2017). Behavioral time scale synaptic plasticity underlies CA1 place fields. Science, 357(6355), 1033–1036. https://doi.org/10.1126/science.aan3846
Cheng, S. (2013). The CRISP theory of hippocampal function in episodic memory. Frontiers in Neural Circuits, 7(May), 88. https://doi.org/10.3389/fncir.2013.00088
Colgin, L. L., Leutgeb, S., Jezek, K., Leutgeb, J. K., Moser, E. I., McNaughton, B. L., & Moser, M.-B. (2010). Attractor-Map Versus Autoassociation Based Attractor Dynamics in the Hippocampal Network. Journal of Neurophysiology, 104(1), 35–50. https://doi.org/10.1152/jn.00202.2010
Corneil, D. S., & Gerstner, W. (2015). Attractor Network Dynamics Enable Preplay and Rapid Path Planning in Maze– like Environments. Advances in Neural Information Processing Systems, 28. https://proceedings.neurips.cc/paper/2015/hash/e515df0d202ae52fcebb14295743063b-Abstract.html
Dunn, S. L. S., Town, S. M., Bizley, J. K., & Bendor, D. (2022). Behaviourally modulated hippocampal theta oscillations in the ferret persist during both locomotion and immobility. Nature Communications, 13(1), Article 1. https://doi.org/10.1038/s41467-022-33507-2
Eliav, T., Geva-Sagiv, M., Yartsev, M. M., Finkelstein, A., Rubin, A., Las, L., & Ulanovsky, N. (2018). Nonoscillatory Phase Coding and Synchronization in the Bat Hippocampal Formation. Cell, 175(4), 1119-1130.e15. https://doi.org/10.1016/j.cell.2018.09.017
Feng, T., Silva, D., & Foster, D. J. (2015). Dissociation between the Experience-Dependent Development of Hippocampal Theta Sequences and Single-Trial Phase Precession. Journal of Neuroscience, 35(12), 4890–4902. https://doi.org/10.1523/JNEUROSCI.2614-14.2015
Frank, L. M., Stanley, G. B., & Brown, E. N. (2004). Hippocampal Plasticity across Multiple Days of Exposure to Novel Environments. Journal of Neuroscience, 24(35), 7681–7689. https://doi.org/10.1523/JNEUROSCI.1958-04.2004
Geisler, C., Robbe, D., Zugaro, M., Sirota, A., & Buzsáki, G. (2007). Hippocampal place cell assemblies are speed-controlled oscillators. Proceedings of the National Academy of Sciences of the United States of America, 104(19), 8149–8154. https://doi.org/10.1073/pnas.0610121104
Grosmark, A. D., & Buzsaki, G. (2016). Diversity in neural firing dynamics supports both rigid and learned hippocampal sequences. Science, 351(6280), 1440–1443. https://doi.org/10.1126/science.aad1935
Hasselmo, M. E., Bodelón, C., & Wyble, B. P. (2002). A Proposed Function for Hippocampal Theta Rhythm: Separate Phases of Encoding and Retrieval Enhance Reversal of Prior Learning. Neural Computation, 14(4), 793–817. https://doi.org/10.1162/089976602317318965
Huxter, J., Burgess, N., & O’Keefe, J. (2003). Independent rate and temporal coding in hippocampal pyramidal cells. Nature, 425(6960), 828–832. https://doi.org/10.1038/nature02058
Jezek, K., Henriksen, E. J., Treves, A., Moser, E. I., & Moser, M.-B. (2011). Theta-paced flickering between place-cell maps in the hippocampus. Nature, 478(7368), 246–249. https://doi.org/10.1038/nature10439
Kennedy, J. P., Zhou, Y., Qin, Y., Lovett, S. D., Sheremet, A., Burke, S. N., & Maurer, A. P. (2022). A Direct Comparison of Theta Power and Frequency to Speed and Acceleration. Journal of Neuroscience, 42(21), 4326–4341. https://doi.org/10.1523/JNEUROSCI.0987-21.2022
Knierim, J. J., & Neunuebel, J. P. (2015). Tracking the flow of hippocampal computation: Pattern separation, pattern completion, and attractor dynamics. Neurobiology of Learning and Memory. http://view.ncbi.nlm.nih.gov/pubmed/26514299
Laptev, D., & Burgess, N. (2019). Neural Dynamics Indicate Parallel Integration of Environmental and Self-Motion Information by Place and Grid Cells. Frontiers in Neural Circuits, 13. https://www.frontiersin.org/articles/10.3389/fncir.2019.00059
Muller, R. U., & Kubie, J. L. (1989). The firing of hippocampal place cells predicts the future position of freely moving rats. The Journal of Neuroscience, 9(12), 4101–4110.
Parra-Barrero, E., Diba, K., & Cheng, S. (2021). Neuronal sequences during theta rely on behavior-dependent spatial maps. ELife, 10, e70296. https://doi.org/10.7554/eLife.70296
Petersen, P. C., & Buzsáki, G. (2020). Cooling of Medial Septum Reveals Theta Phase Lag Coordination of Hippocampal Cell Assemblies. Neuron, 0(0). https://doi.org/10.1016/j.neuron.2020.05.023
Priestley, J. B., Bowler, J. C., Rolotti, S. V., Fusi, S., & Losonczy, A. (2022). Signatures of rapid plasticity in hippocampal CA1 representations during novel experiences. Neuron. https://doi.org/10.1016/j.neuron.2022.03.026
Rolls, E. T. (2017). A scientific theory of Ars Memoriae: Spatial view cells in a continuous attractor network with linked items. Hippocampus, 27(5), 570–579. https://doi.org/10.1002/hipo.22713
Romani, S., & Tsodyks, M. (2015). Short-term plasticity based network model of place cells dynamics. Hippocampus, 25(1), 94–105. https://doi.org/10.1002/hipo.22355
Samsonovich, A., & McNaughton, B. L. (1997). Path integration and cognitive mapping in a continuous attractor neural network model. The Journal of Neuroscience, 17(15), 5900–5920.
Schmidt, R., Diba, K., Leibold, C., Schmitz, D., Buzsáki, G., & Kempter, R. (2009). Single-Trial Phase Precession in the Hippocampus. Journal of Neuroscience, 29(42), 13232–13241. https://doi.org/10.1523/JNEUROSCI.2270-09.2009
Sheffield, M. E. J., Adoff, M. D., & Dombeck, D. A. (2017). Increased Prevalence of Calcium Transients across the Dendritic Arbor during Place Field Formation. Neuron, 96(2), 490-504.e5. https://doi.org/10.1016/j.neuron.2017.09.029
Shen, J., Barnes, C. A., McNaughton, B. L., Skaggs, W. E., & Weaver, K. L. (1997). The Effect of Aging on Experience-Dependent Plasticity of Hippocampal Place Cells. Journal of Neuroscience, 17(17), 6769–6782. https://doi.org/10.1523/JNEUROSCI.17-17-06769.1997
Sławińska, U., & Kasicki, S. (1998). The frequency of rat’s hippocampal theta rhythm is related to the speed of locomotion. Brain Research, 796(1), 327–331. https://doi.org/10.1016/S0006-8993(98)00390-4
Tanni, S., de Cothi, W., & Barry, C. (2022). State transitions in the statistically stable place cell population correspond to rate of perceptual change. Current Biology. https://doi.org/10.1016/j.cub.2022.06.046
Wang, Y., Romani, S., Lustig, B., Leonardo, A., & Pastalkova, E. (2015). Theta sequences are essential for internally generated hippocampal firing fields. Nature Neuroscience, 18(2), 282–288. https://doi.org/10.1038/nn.3904
Wikenheiser, A. M., & Redish, A. D. (2015). Hippocampal theta sequences reflect current goals. Nature Neuroscience, 18(2), 289–294.
Wilson, M. A., & McNaughton, B. L. (1993). Dynamics of the hippocampal ensemble code for space. Science, 261(5124), 1055–1058. https://doi.org/10.1126/science.8351520
